# Point-of-Care Capillary Compared to Venous Bilirubin Measurement in Neonates

**DOI:** 10.1007/s12291-024-01194-z

**Published:** 2024-03-10

**Authors:** Dimitrios Rallis, Maria Baltogianni, Elena Maragoudaki, Paschalina Tseklazi, Konstantina Kapetaniou, Vasileios Giapros

**Affiliations:** 1 Neonatal Intensive Care Unit, School of Medicine, University of Ioannina, Stavrou Niarchou Avenue, 45110 Ioannina, Greece; 2 Department of Paediatrics, School of Medicine, University of Ioannina, Stavrou Niarchou Avenue, 45110, Ioannina, Greece

**Keywords:** Diagnostic test, Hyperbilirubinemia, Jaundice, Phototherapy, Point-of-care

## Abstract

We aimed to evaluate the agreement between the point-of-care (POC) capillary bilirubin measurement with POC venous samples and the reference laboratory measurement. We conducted a prospective comparative study, including neonates  ≥ 34 weeks of gestational age, and ≥ 72 h of age. The agreement between POC (Calmark Neo-Bilirubin, Sommargatan, Karlstad, Sweden) capillary, POC venous, and laboratory venous bilirubin was examined with the Bland–Altman plot and the Passing-Bablok regression analyses. The mean bilirubin was 13.54 (2.79) mg/dL in the POC capillary samples, 13.45 (2.69) mg/dL in the POC venous samples, and 12.68 (2.33) mg/dL in reference samples. Bland–Altman plots showed optimal agreement between the POC capillary and venous methods, and with the reference venous method. The bias between the POC capillary and venous methods was 0.094 [levels of agreement (− 3.118)− 3.306], between the POC capillary and the reference venous methods 0.865 [levels of agreement (− 2.283)− 4.014], and between the POC venous and the reference venous methods 0.771 [levels of agreement (− 1.814)− 3.357]. The POC capillary and venous bilirubin levels were in optimal agreement with each other, and with the reference venous measurements, supporting the POC Calmark Neo-Bilirubin capillary measurement as an alternative for a less-invasive, more rapid evaluation of bilirubin.

## Introduction

Jaundice, a common neonatal condition, is caused by high levels of bilirubin in the blood [1, 2]. In neonates, indirect hyperbilirubinemia is present in nearly 80% of neonates and is almost always benign; however, monitoring of bilirubin levels is necessary to assess the need for treatment [1, 3].

Typically, the reference method for bilirubin measurement requires the measurement of blood serum samples in a laboratory using chemical reactions [4]. The classic laboratory reaction of the diazo method uses a reagent that measures the direct and total bilirubin, while the indirect bilirubin is then calculated from the difference between the two [5, 6]. This method has the disadvantages of requiring pipetting and using several reagents, in addition to the long time taken to complete the exam [5, 6]. The results might also be influenced by hemolysis or high hematocrit, which is a common condition in neonates within the first days of life [7]. Although blood can be sampled routinely from neonates, the procedure usually requires sampling a considerable amount of blood which is relatively painful and leads to neonatal discomfort. Bilirubin values may also vary depending on the site of collection (e.g., when capillary and venous samples are compared) [8, 9]. Whether capillary-derived values underestimate or overestimate their venous counterparts has been controversial [10–12].

Point-of-care (POC) analyzers can measure indirect bilirubin requiring only a minimal amount of blood and providing the results within minutes from the sampling. To date, there are only a few studies investigating the agreement between the laboratory and the POC methods as alternatives for bilirubin measurement [13–17]. The POC Calmark Neo-Bilirubin (Calmark, Sommargatan, Karlstad, Sweden) is a medical diagnostic device that allows efficient, fast, and easy measurement of bilirubin levels; however, the accuracy of the device has not been evaluated in capillary samples.

The present study aimed to evaluate the agreement between POC capillary and venous bilirubin measurements, as well as the agreement between both POC measurements with the reference laboratory venous bilirubin measurements.

## Material and Methods

### Study Design, Parameters, and Data Collection

We conducted a prospective comparative study in the University Hospital of Ioannina, Greece, during an eight-month period, 10/2022–5/2023. The Ethics Committee of the Institution approved the study (No: 950) and written consent from all parents was obtained at enrollment.

Neonates were eligible for enrollment if they were over 34 weeks of gestational age, over 72 h of age, and required evaluation of bilirubin levels as per clinical practice either in the Neonatal Unit or the Maternal Ward.

### Blood Sampling

In each neonate, a venous blood sample of 250–500 μl was obtained for the measurement of bilirubin levels in the biochemical laboratory of our institution, as per routine clinical practice (reference measurement). Any decisions regarding the clinical management of each neonate were based on the laboratory bilirubin measurement only. The venous blood sample was obtained from each neonate by clean venipuncture with a 21-French gauge needle, allowing free dripping of blood into a BD Vacutainer® SST II Advance tube (BD-Beliver Industrial, Plymouth, UK). A minimum amount of 70 μl from the same venous blood sample was used for the evaluation of bilirubin with the POC analyzer, which was performed in the settings of the Neonatal Unit. At the same time, 70 μl of a capillary blood sample was obtained by heel prick into a similar collecting tube. The heel of the neonate was adequately warmed before pricking, avoiding the squeezing of blood and allowing the free drainage of blood. The capillary bilirubin measurement was performed with the POC analyzer in the setting of the Neonatal Unit, as well. We constantly analyzed the venous samples first, followed by the capillary samples, to keep a standardized analysis, and have potential tracking of the results available, if needed.

### Instruments

#### Laboratory Analyzer

The Beckman Coulter AU5820 analyzer is used in our laboratory for bilirubin (total and direct) measurement (Beckman Coulter, Inc., Brea, CA, USA). A stabilized diazonium salt, 3,5-dichlorophenyldiazonium tetrafluoroborate, reacts with bilirubin to form azobilirubin which absorbs at 570/660 nm. Caffeine and a surfactant are used as reaction accelerators. The absorbance at 570/660 nm is proportional to the bilirubin concentration in the sample. A separate serum blank is performed to eliminate endogenous serum interferences. Direct bilirubin couples directly with a diazonium salt of 3,5-dichloroaniline in an acid medium to form azobilirubin. The direct bilirubin in serum is directly proportional to the color development of azobilirubin which is measured bichromatically at 570/660 nm. According to the manufacturer, the criteria for non-significant interference is recovery within 10% of the initial value, and are estimated for hemolysis to a non-significant interference up to 500 mg/dL hemolysate for the total and up to 10 mg/dL for the direct bilirubin, and for lipemia to a non-significant interference up to 500 mg/dL intralipid for the total and up to 300 mg/dL for the direct bilirubin.

#### Point-of-Care Analyzer

The POC Calmark Neo-Bilirubin analyzer is tested and controlled according to the International Electrotechnical Commission 61010–2-101 and fulfills the requirements for in vitro diagnostics Medical Device Directive 98/79/EC. The test cassette is constituted by four filters with different functions. The first two filters which are in contact with the blood are responsible for the blood filtration. Further, the plasma is separated and migrated through the third filter into the fourth (detection filter) by lateral flow. A color appears in the detection filter. A camera, inside the instrument, takes photos during the test. The color in the detection filter is further analyzed and converted through software into the corresponding numeric value of the bilirubin concentration in the blood sample and presented on the screen. The measuring concentration range of bilirubin is 9–29 mg/dL. The POC analyzer can analyze samples with hemolysis up to 2 g/L and level of hematocrit up to 60%.

The within-run precision using 3 different blood samples and 20 replicates according to the Clinical and Laboratory Standards Institute document EP05-A: Evaluation of Precision of Quantitative Measurement Procedures (CLSI EP5-A), has a coefficient of variation (CV) of 6.9% for bilirubin < 9 mg/dL, 5.2% for 9–15 mg/dL, and 4.4% for > 15 mg/dL. Imprecision was estimated over 20 consecutive working days for commercially available bilirubin controls according to the CLSI EP5-A to CV of 14.0% for low control, and 11.8% for medium control. Bias has been calculated and grouped into three concentration groups, at 12.9% for bilirubin < 9 mg/dL, 8.8% for 9–15 mg/dL, and 4.0% for > 15 mg/dL.

#### Data Collection

The perinatal data including gestational age, sex, birth weight, delivery method, and the clinical outcomes of the neonates were collected.

### Statistical Analysis

Perinatal data and clinical outcomes were expressed with descriptive statistics. Continuous variables were expressed as mean (standard deviation, SD) or median (interquartile range), whereas categorical variables as n (percentage, %). The normality of the distribution was examined with the Kolmogorov-Smirnoff test or the Kruskal–Wallis test, as appropriate.

The agreement between the POC capillary and venous bilirubin measurements, and between both POC measurements and the reference laboratory venous bilirubin measurements was examined with the Bland–Altman plot. The Bland–Altman method of agreement between two quantitative measurements determines the bias (mean difference between the reference and alternative methods) and limits of agreement (LOA) resulting from the bias ± 1.96 times the SD of the bias. The systematic and the proportional bias were examined with the Passing-Bablok regression analyses, evaluating the intercept (a) and the slope (b) with their 95% confidence intervals (CI), respectively, of the regression formula: y = a + b*x (where y is the tested, and y is the reference method). If 95% CI for intercept includes value zero, there is no constant difference between the two methods. Accordingly, if 95% CI for slope includes value one, there is no proportional difference between the two methods. Statistical significance was defined for p < 0.05. The total error between the POC capillary and venous bilirubin measurements and the reference laboratory venous bilirubin measurements was examined and evaluated according to the total allowable error suggested by Clinical Laboratory Improvement Amendments of 1988 (CLIA) Proficiency Testing regulations, that was for bilirubin ± 20%, or 0.4 mg/dL [18]. Finally, we examined whether there was any difference between the POC and the reference methods in detecting neonates with bilirubin levels necessitating phototherapy. We calculated the sensitivity, specificity, positive predictive value (PPV), and negative predictive value (NPV) of both POC capillary and venous methods for the correct identification of neonates necessitating phototherapy. The receiver operating characteristic (ROC) curve analyses and the Area Under the Curve (AUC) were constructed.

A power analysis revealed that to achieve a power of 80% and a type I error α of 0.05, considering the study design, a pair of more than 45 samples was adequate to detect the desired absolute difference of bilirubin levels between any two methods of 10%. Data were analyzed with Microsoft Excel 365 (Microsoft, Redmond, WA, USA), and SPSS statistical software version 25.0 (SPSS Inc., Chicago, IL, USA). Plots were done using Prism 9.0c (GraphPad Software, La Jolla, CA, USA).

## Results

During the study period, we enrolled 54 neonates, obtaining 84 pairs of venous and capillary blood samples. Neonates were of a mean of 37.1 (1.8) weeks of gestational age and birth weight of 2931 (536) g. Among them, 30 (56%) were males, 32 (59%) were born by cesarean section, and 45 (83%) neonates were singleton. Eight (15%) neonates were admitted for respiratory distress, whereas six (8%) neonates received phototherapy. The mean hematocrit level was 48.6 (4.2) %, and the mean hemoglobin 16.7 (1.4) g/dL.

**Fig. 1:**
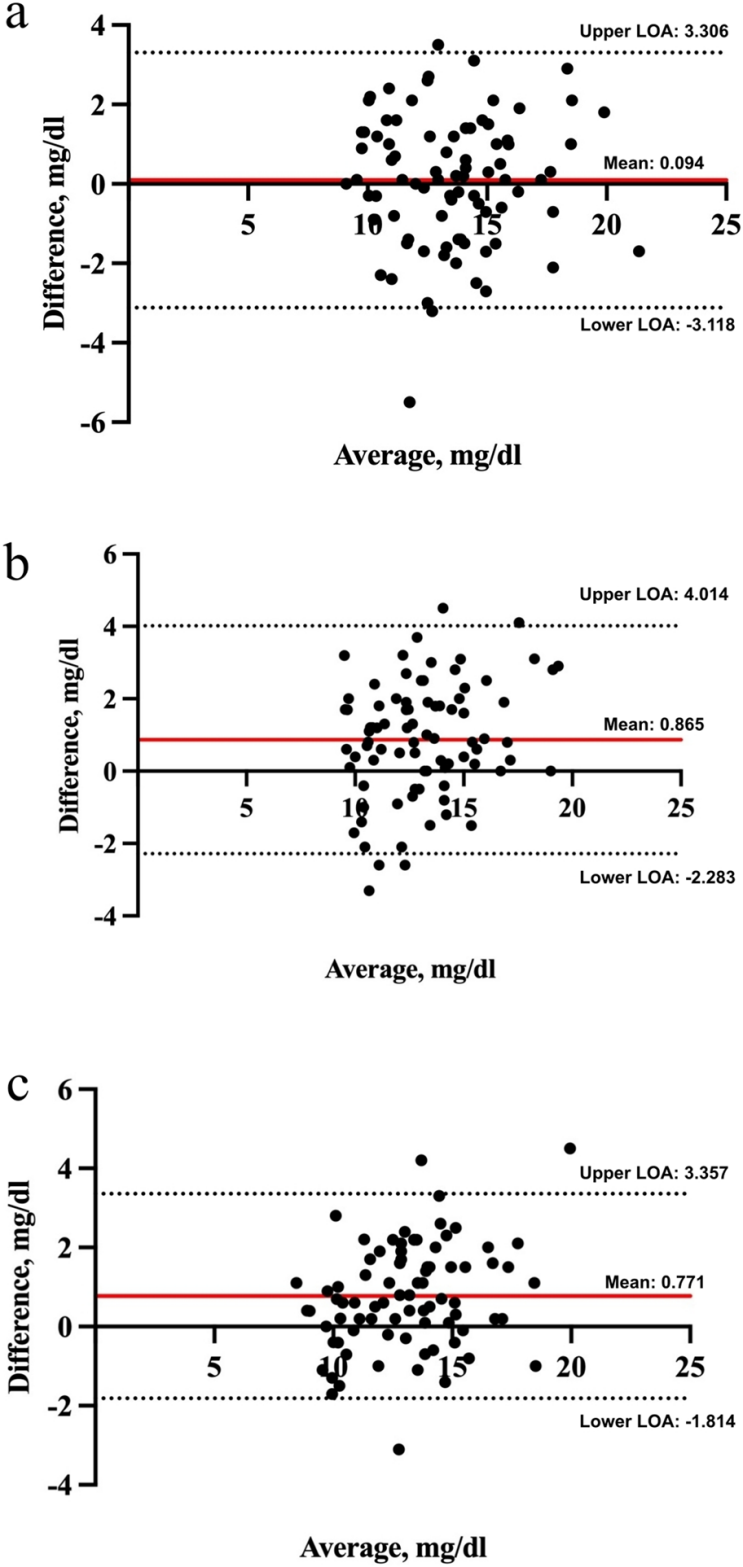
**a** Bland–Altman plot of the POC capillary and POC venous bilirubin measurements. **b** Bland–Altman plot of the POC capillary and the reference venous bilirubin measurements. c Bland–Altman plot of the POC venous and the reference venous bilirubin measurements. POC, point-of-care; LOA, limits of agreement

**Fig. 2:**
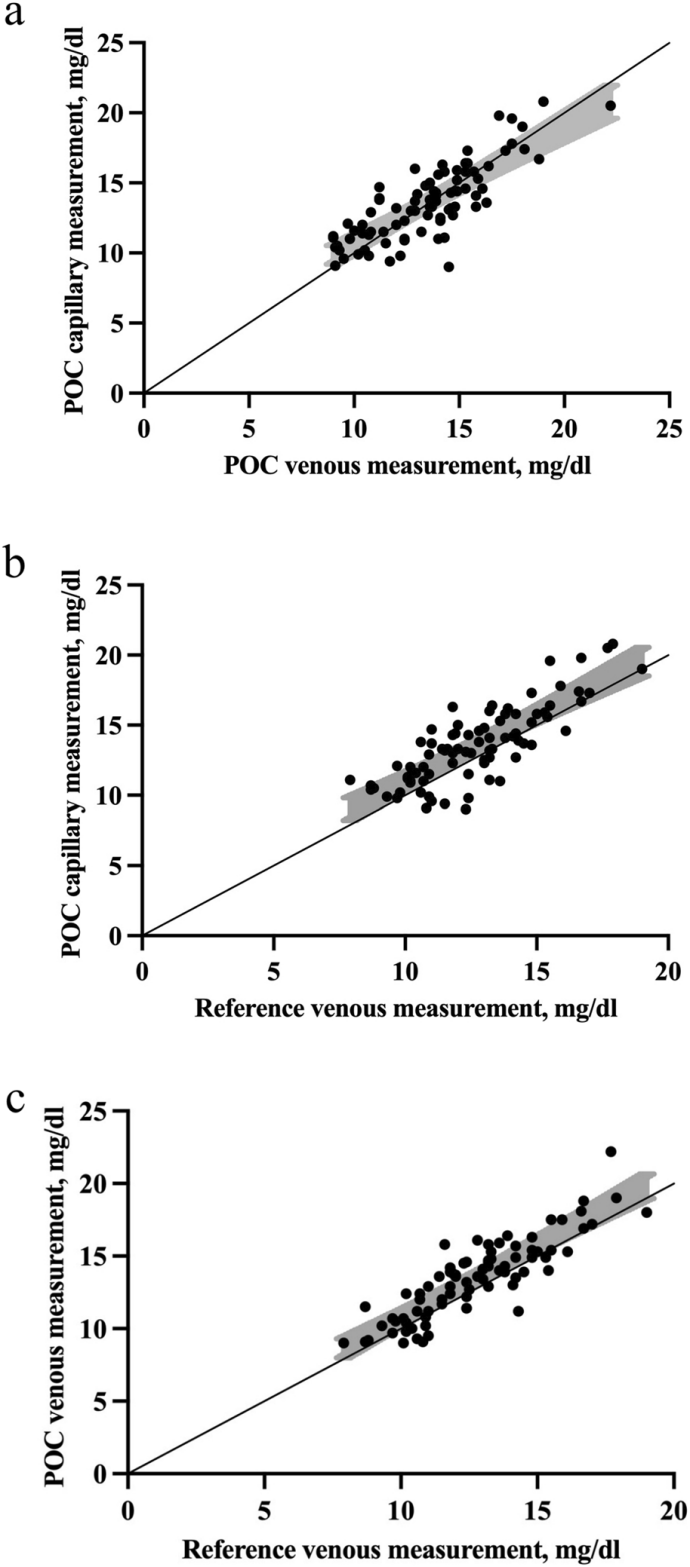
**a** Correlation between the POC capillary and POC venous bilirubin measurements. **b**. Correlation between the POC capillary and the reference venous bilirubin measurements. **c**. Correlation between the POC venous and the reference venous bilirubin measurements. POC, point-of-care

The mean bilirubin was 13.54 (2.79) mg/dL in the POC capillary samples, 13.45 (2.69) mg/dL in the POC venous samples, and 12.68 (2.33) mg/dL in reference venous samples. Bland–Altman plots showed optimal agreement between the POC capillary and venous methods, and between the POC methods, both the venous and the capillary, and the reference venous method (Fig. 1). The bias between the POC capillary and POC venous methods was 0.094 [LOA (-3.118)-3.306], between the POC capillary and the reference venous methods was 0.865 [LOA (-2.283)-4.014], and between the POC venous and the reference venous and was 0.771 [LOA (-1.814)-3.357] (Table 1). The residual plots of the distribution of the differences between POC and reference methods around the fitted regression line of the reference method are depicted in Fig. 2.

**Table 1:** Bland–Altman analysis between the two tested and the reference methods

	Mean difference	SD difference	Range difference	LOA
	(bias)	(bias)	(bias)	
POC capillary compared to POC venous method, mg/dl 0.094	0.094	1.638	(− 5.5)− 3.5	(− 3.118)− 3.306
POC capillary compared to reference venous method, mg/dl 0.865	0.865	1.606	(− 3.3)− 4.5	(− 2.283)− 4.014
POC venous compared to reference venous method, mg/dl 0.771	0.771	1.319	(− 3.1)− 4.5	(− 1.814)− 3.357

SD, standard deviation; LOA, limits of agreement; POC, point-of-care

**Fig. 3:**
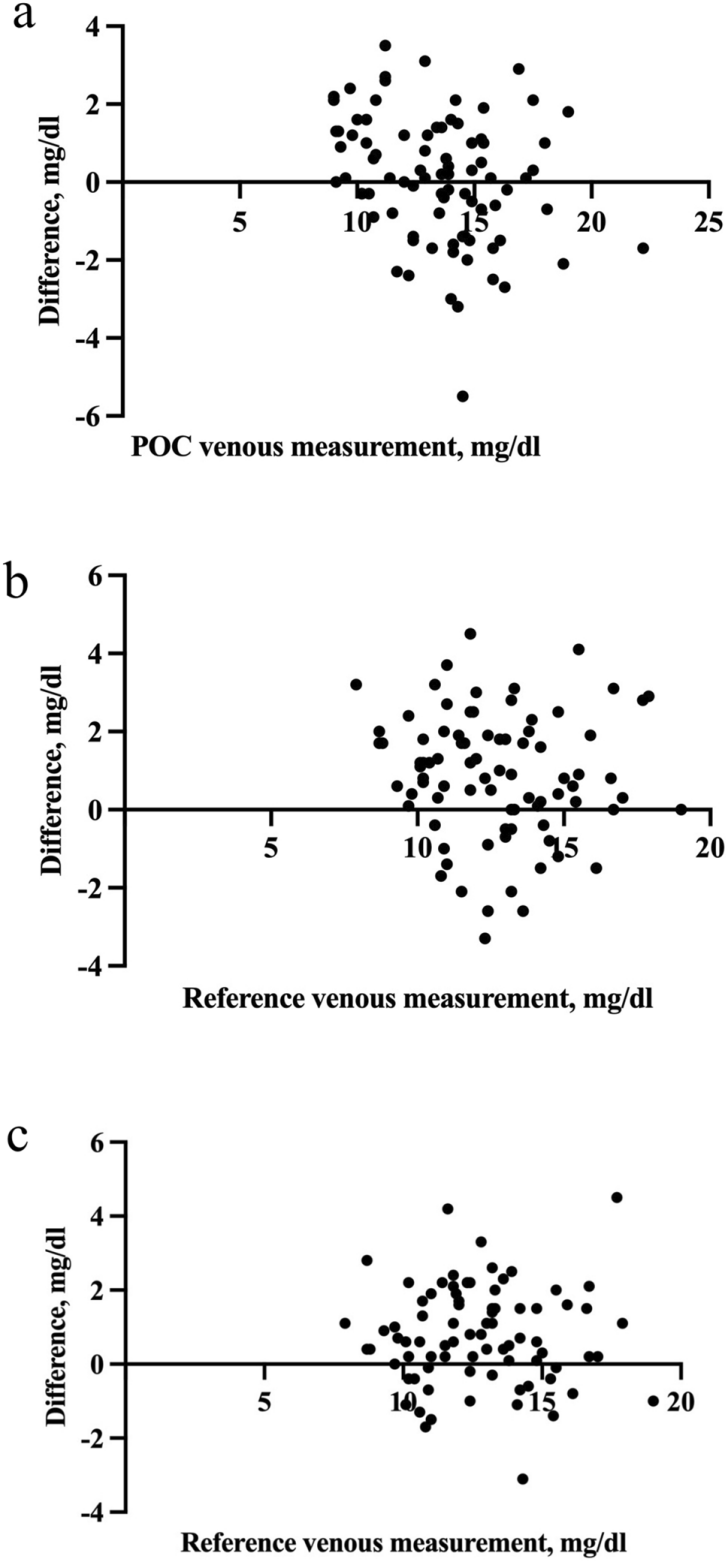
**a**. Residual plot of difference between the POC capillary and POC venous bilirubin measurements around the POC venous measurement. **b**. Residual plot of difference between the POC capillary and the reference venous bilirubin measurements around the reference venous measurement. **c**. Residual plot of difference between the POC venous and the reference venous bilirubin measurements around the reference venous measurement. POC, point-of-care

In Passing-Bablok regression analyses, as shown in Table 2 and Fig. 3, we found correlation coefficients *(r)* 0.781 between the POC capillary and POC venous methods, 0.777 between the POC capillary and the reference venous methods, and 0.861 between the POC venous and the reference venous methods. The slope (b) of the regression line was 1.025 between POC capillary and POC venous methods, 1.136 between POC capillary and reference venous methods, and 1.187 between POC venous and reference venous methods. The intercept (a) between the POC capillary and POC venous methods, the POC capillary and the reference venous, and the POC venous and the reference venous methods were -3.327, -6.681, and -13.343, respectively. The 95% CI of slopes showed that the amount of proportional bias between the POC capillary and POC venous methods, between the POC capillary and the reference venous, and the POC venous and the reference venous methods was not significant. Moreover, the 95% CI of intercepts revealed that the systematic bias between the two POC methods, and between the two POC methods and the reference methods was also not significant (Table 2).

**Table 2:** Passing-Bablock regression analysis between the two tested and the reference methods

	r	95% CI	Slope (b)	Slope 95% CI	Intercept (a) (mg/dl)	Intercept 95% CI (mg/dl)
POC capillary compared to POC venous method	0.781	0.678–0.855	1.025	0.890–1.208	− 3.327	(− 26.833)− 12.771
POC capillary compared to reference venous method	0.777	0.672–0.851	1.136	0.937–1.396	− 6.681	(− 37.433)− 17.312
POC venous compared to reference venous method	0.861	0.790–0.909	1.187	0.977–1.450	− 13.343	(− 46.25)− 10.522

CI, confidence intervals; POC, point-of-care

The total error between the POC capillary measurements and the reference venous measurements was 11.1%, while between the POC venous measurements and the reference laboratory venous measurements 8.7%; both were acceptable and lower than the total allowable error of 20%.

**Fig. 4:**
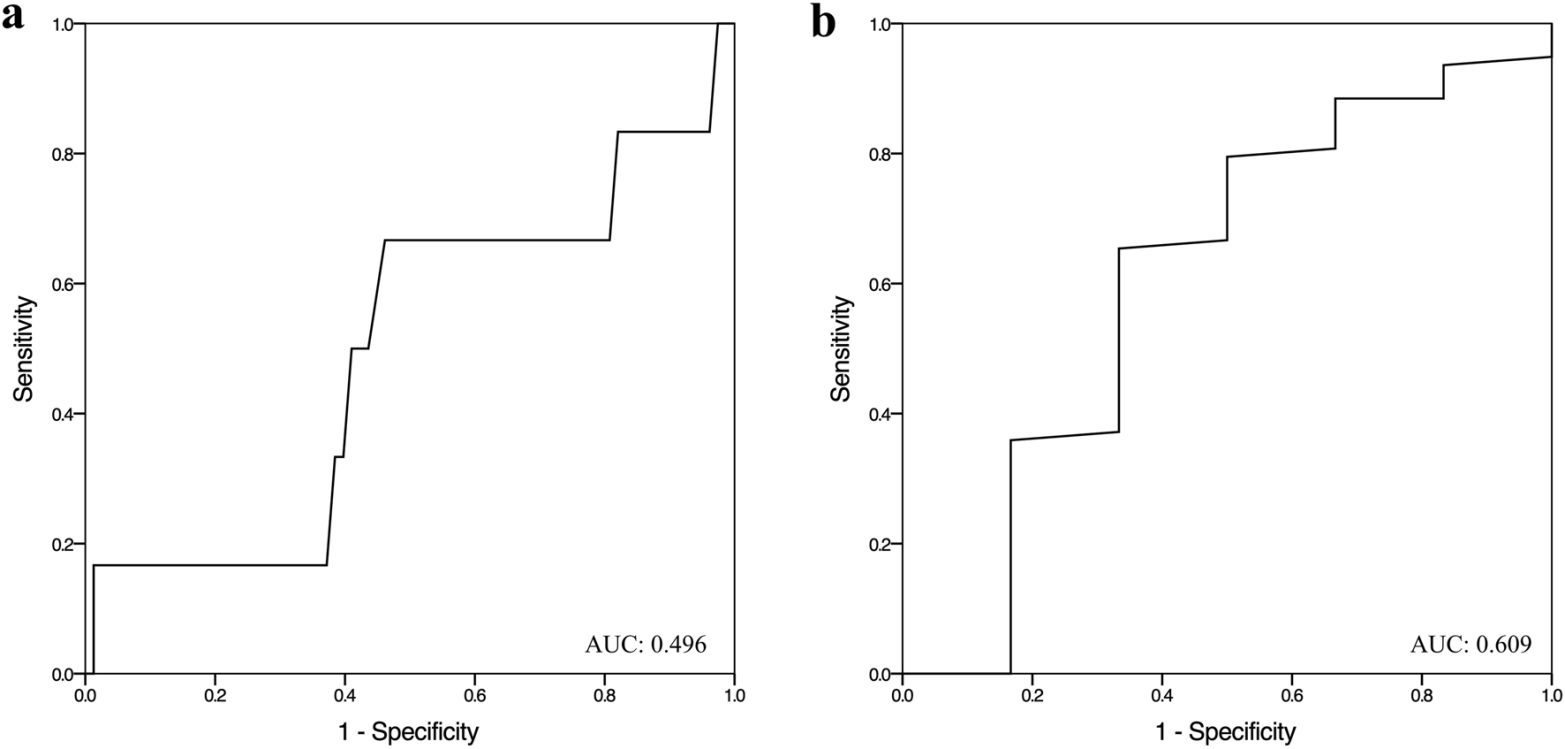
**a**. The receiver operating characteristic curve analyses and the Area Under the Curve of the POC capillary method for the correct identification of neonates necessitating phototherapy. **b**. The receiver operating characteristic curve analyses and the Area Under the Curve of the POC venous method for the correct identification of neonates necessitating phototherapy POC, point-of-care; AUC, Area Under the Curve

Regarding the accuracy of the POC methods in detecting neonates with a bilirubin levels above the phototherapy threshold, we found that the six neonates who required phototherapy [mean bilirubin cutoff 12.0 (2.82) mg/dL] were all detected with both POC venous and capillary methods, while both POC methods each detected six additional neonates (7%) as requiring phototherapy [mean capillary bilirubin cutoff 14.75 (3.93) mg/dL, mean venous bilirubin cutoff 13.67 (3.25) mg/dL]. The positive and negative predictive values of the POC capillary method for a bilirubin cutoff of 13.8 mg/dL was 1.44 and 0.69 respectively, and the positive and negative predictive values of the POC venous method for a bilirubin cutoff of 12.4 mg/dL was 1.96 and 0.51, respectively (Table 3). The ROC analysis is presented in Fig. 4.

**Table 3:** The sensitivity, specificity, positive predictive value and negative predictive value of the point-of-care capillary and venous methods

	Bilirubin cutoff (mg/dL)	Sensitivity	Specificity	Positive predictive value	Negative predictive value
*Point-of-care*					
Capillary method	13.8	0.66	0.54	1.44	0.69
Venous method	12.4	0.65	0.67	1.96	0.51

## Discussion 

The findings of this study suggested that the bilirubin values that were measured in capillary blood samples with the POC analyzer were in optimal agreement with POC venous samples, as well as with the reference venous samples. We also found that both POC venous and capillary measurements had an optimal accuracy in not missing neonates with a bilirubin level above the phototherapy threshold. Based on our findings, the POC analyzer was found to be a useful tool for an instant capillary bilirubin measurement. This device could thus be used in the primary care settings without necessitating the measurement of bilirubin in a hospital which might be time-consuming and anxiety-inducing to parents.

In accordance with our study, previous investigators who examined the agreement between different laboratory and POC bilirubin analyzers also found an optimal agreement between the methods [13, 14]. Grohmann et al. compared nine frequently used methods for bilirubin determination, including cutaneous, venous POC analyzers, and venous laboratory analyzers, and found an optimal agreement between the comparison values for all nonchemical photometric devices [13]. Also, Leite et al. found an optimal agreement between the laboratory colorimetric method through the traditional venous blood collection and the direct spectrophotometry of capillary blood [14].

Moreover, the relationship between capillary and venous measurements is of significant importance in clinical practice. Evidence from previous studies has suggested a difference between the laboratory serum samples and the capillary measurements, reporting that capillary levels tend to overestimate the laboratory levels by 0.558 mg/dL or up to 7% [8, 15, 19]. On the other hand, capillary samples might underestimate venous measurements, by a difference between 0.91 mg/dL to 2.73 mg/dL in favor of laboratory venous measurements, especially when bilirubin levels exceed 15 mg/dL [9, 14, 20, 21]. Our study found that both capillary and venous POC bilirubin measurements were slightly higher than the reference venous measurement, with an average difference of 0.865 mg/dL between POC capillary and the reference venous methods, and an average of 0.771 mg/dL between the POC venous and the reference venous measurements. Between the two POC measurements, we found a minimum average difference of 0.094 mg/dL in favor of the capillary sample. This difference between the two POC and the reference measurements might be attributed to technical issues. The POC samples were measured immediately upon collection, whereas the laboratory sample was measured with a reasonable delay due to the transfer to the laboratory. Thus, a potential effect of the light on the isomerization of bilirubin could not be excluded [8, 9]. Also, the results of the POC instrument are not influenced by any substantial hemolysis of the sample, contrary to laboratory analysis. That might also explain the fact that both POC measurements were similar and equally different from the laboratory method.

The limitations of our study arise firstly from its single-center design; thus, our results need further evaluation before generalization. Also, we examined only neonates who were born above 34 weeks of gestational age; therefore, we could not evaluate the agreement between the reference and the POC methods in preterm neonates below 34 weeks of gestational age.

In conclusion, the POC Calmark Neo-Bilirubin capillary bilirubin levels were in optimal agreement with the POC venous levels, and both POC measurements were in optimal agreement with the reference measurements. POC measurements slightly overestimated bilirubin levels compared to the reference venous measurements. Finally, both POC venous and capillary measurements had optimal accuracy in not missing neonates with bilirubin levels above the phototherapy threshold.

## Abbreviations

CI: Confidence intervals
LOA: Limits of agreement
SD: Standard deviation

## References

[1] Kemper AR, Newman TB, Slaughter JL, Maisels MJ, Watchko JF, Downs SM, et al. Clinical practice guideline revision: management of hyperbilirubinemia in the newborn infant 35 or more weeks of gestation. Pediatrics. 2022. 10.1542/peds.2022-058859. 35927462 10.1542/peds.2022-058859

[2] Slaughter JL, Kemper AR, Newman TB. Technical report: diagnosis and management of hyperbilirubinemia in the newborn infant 35 or more weeks of gestation. Pediatrics. 2022. 10.1542/peds.2022-058865. 35927519 10.1542/peds.2022-058865

[3] Bhutani VK, Stark AR, Lazzeroni LC, Poland R, Gourley GR, Kazmierczak S, et al. Predischarge screening for severe neonatal hyperbilirubinemia identifies infants who need phototherapy. J Pediatr. 2013;162:477-82.e1. 10.1016/j.jpeds.2012.08.022. 23043681 10.1016/j.jpeds.2012.08.022

[4] de Cordova CM, Nogara MS, de Cordova MM. Interference on the laboratory measurement of bilirubin: the effect of in vitro interactions. Clin Chim Acta. 2009;407:77–9. 10.1016/j.cca.2009.06.037. 19580796 10.1016/j.cca.2009.06.037

[5] Sykes E, Epstein E. Laboratory measurement of bilirubin. Clin Perinatol. 1990;17:397–416. 2196137

[6] Muraca M, Blanckaert N. Liquid-chromatographic assay and identification of mono- and diester conjugates of bilirubin in normal serum. Clin Chem. 1983;29:1767–71. 6616822

[7] Brunori P, Masi P, Faggiani L, Villani L, Tronchin M, Galli C, et al. Evaluation of bilirubin concentration in hemolysed samples, is it really impossible? The altitude-curve cartography approach to interfered assays. Clin Chim Acta. 2011;412:774–7. 10.1016/j.cca.2011.01.010. 21238446 10.1016/j.cca.2011.01.010

[8] Eidelman AI, Schimmel MS, Algur N, Eylath U. Capillary and venous bilirubin values: they are different–and how! Am J Dis Child. 1989;143:642. 10.1001/archpedi.1989.02150180020007. 2729205 10.1001/archpedi.1989.02150180020007

[9] Leslie GI, Philips JB 3rd, Cassady G. Capillary and venous bilirubin values Are they really different? Am J Dis Child. 1987;141:1199–200. 10.1001/archpedi.1987.04460110069024. 3673971 10.1001/archpedi.1987.04460110069024

[10] Maisels MJ. Capillary vs venous bilirubin values. Am J Dis Child. 1990;144:521–2. 10.1001/archpedi.1990.02150290015011. 2330914 10.1001/archpedi.1990.02150290015011

[11] Newman TB, Maisels MJ. The bilirubin debate. Pediatrics. 1992;90:132. 1614770

[12] Newman TB, Klebanoff MA, Maisels MJ. Bilirubin problem–the debate continues. Pediatrics. 1996;98:165–6. 8668399

[13] Grohmann K, Roser M, Rolinski B, Kadow I, Muller C, Goerlach-Graw A, et al. Bilirubin measurement for neonates: comparison of 9 frequently used methods. Pediatrics. 2006;117:1174–83. 10.1542/peds.2005-0590. 16585313 10.1542/peds.2005-0590

[14] Leite M, Granato Vde A, Facchini FP, Marba ST. Comparison of transcutaneous and plasma bilirubin measurement. J Pediatr (Rio J). 2007;83:283–6. 10.2223/JPED.1619. 17508094 10.2223/JPED.1619

[15] Cristina AC. Determining the correlation and accuracy of three methods of measuring neonatal bilirubin concentration: serum capillary and transcutaneous bilirubin. Biomed J Sci Tech Res. 2017. 10.26717/bjstr.2017.01.000286.

[16] Gu D, Wang Y, Ren B, Wang L, Zhang K, Yuan Y. Comparison of three routine methods for the measurement of serum bilirubin in a China laboratory. Clin Lab. 2018;64:1485–90. 10.7754/Clin.Lab.2018.180333. 30274008 10.7754/Clin.Lab.2018.180333

[17] Wang L, Albert AY, Jung B, Hadad K, Lyon ME, Basso M. Limitations and opportunities of whole blood bilirubin measurements by GEM premier 4000(R). BMC Pediatr. 2017;17:92. 10.1186/s12887-017-0842-8. 28356083 10.1186/s12887-017-0842-8 PMC5372304

[18] Medicare M. CLIA programs; regulations implementing the Clinical Laboratory Improvement Amendments of 1988 (CLIA)–HCFA. Final rule with comment period. Fed Regist. 1992;57:7002–186. 10170937

[19] Langbaum ME, Pomerance JJ, Farber SJ, Rosenthal P. Comparison of arterial and capillary bilirubin values in neonates with arterial lines. J Pediatr. 1993;123:794–6. 10.1016/s0022-3476(05)80863-7. 8229494 10.1016/s0022-3476(05)80863-7

[20] Franz AR, Pohlandt F, Bode H, Mihatsch WA, Sander S, Kron M, et al. Intrauterine, early neonatal, and postdischarge growth and neurodevelopmental outcome at 5.4 years in extremely preterm infants after intensive neonatal nutritional support. Pediatrics. 2009;123:e101–9. 10.1542/peds.2008-1352. 19117831 10.1542/peds.2008-1352

[21] Mussavi M, Niknafs P, Bijari B. Determining the correlation and accuracy of three methods of measuring neonatal bilirubin concentration. Iran J Pediatr. 2013;23:333–9. 23795258 PMC3684480

